# Annotations

**Published:** 1900-09-15

**Authors:** 


### Annotations.

### Water for Moving Troops.

In a letter which appeared in the *Standard* last Monday, Dr. Leigh Canney discussed the question of the amount of transport which would be required to provide an army with an efficient water-purifying apparatus. It is no new question. The fact has to be faced that no army can ever safely trust to such water as may be picked up on the way, and that, although the transport of filters or sterilisers may be difficult, and may at first sight seem impossible, it is not so difficult and the delay entailed by it can seldom be so disastrous as the difficulty and delay entailed by having a large proportion of the men laid up with dysentery and typhoid fever just when they are wanted. Dr. Canney estimates that for an army of 50,000 men, such as moved from the Modder to Paardeberg and thence to Bloemfontein, the apparatus required to furnish a proper supply of filtered water would have weighed about 11 tons, and to supply boiled water about 22 tons, together with petroleum and spirit as fuel. On the other hand, the boiling apparatus would, he says, have abolished the need for about 4,000 bottles, so that, on the whole, the boiling apparatus would only have added about two tons weight, together with that of 150 gallons of petroleum and 20 gallons of spirit to the 500 tons of stores daily required by this army, an addition requiring an amount of transport absolutely trivial compared with what would be required to move a score or two of fever cases with their necessary attendants. In regard to the march to Bloemfontein, however, it may turn out that, whatever appliances had been provided, they might have been left behind. It has to be recognised that a commander may find it necessary at times to run risks. It is hard, and seems cruel, but military exigencies may demand the sacrifice of life to attain a given end, and in this particular case, if it was found necessary for military reasons to cut down baggage to the smallest proportion; and if for that reason the general in command, knowing the risk, deliberately ordered the men to advance without any means of purifying water, we have nothing to say, any more than we should if he deliberately ordered a regiment to hold a kopje for so many hours with a certain useful object, knowing it would be decimated in so doing. The risk is taken, and the result is to be estimated on purely military grounds. But in regard to the preliminary arrangements made by the War Office for ensuring that the water supplied to the whole of the great army in South Africa should be pure and free from disease-taint, that is another matter, and must, we imagine, form one of the subjects as to which the Commission which has been sent out will make careful inquiries. We do not wish to suggest that there has been default, but in the present state of medical knowledge in regard to the etiology of typhoid fever, we can hardly imagine that the military authorities would be held blameless if it should turn out that the army was sent into such a typhoid country as South Africa without an absolutely reliable means of purifying water. Such means must of necessity be costly and cumbersome, but far less so than providing for thousands of cases of typhoid fever.

### Accommodation for Plague Cases in London.

At the meeting of the Metropolitan Asylums Board held last Saturday reference was made to the accommodation to be provided for any such sporadic or suspected cases of plague as might occur in the metropolis, and Mr. Duncombe Mann, the clerk to the Managers, submitted a memorandum showing what had already been done in the matter. He said that, inquiries having been addressed to him by certain of the metropolitan medical officers of health as to whether any arrangements had been made for the reception of plague cases into their hospitals, he had directed the superintendents of the ambulance stations to remove cases certified to be suffering from plague to the South-Eastern Hospital, and had informed the medical officers accordingly. He said that for some time past, at the suggestion of Dr. Downes, of the Local Government Board, a certain number of beds at the South-Eastern Hospital, which was the nearest to the docks, had been set aside and kept in readiness for the reception of any possible cases of plague, and he had now directed the medical superintendent to proceed immediately with the cleansing, disinfection, and other necessary preparations of a second ward at that hospital for the reception of such cases if they should come in. This, he said, had been duly notified to Dr. Downes, who had promised that, in the event of the Managers unfortunately being called upon to receive cases of plague at their hospitals, the Local Government Board would take such measures as might be necessary to legalise their action in the matter. It may be well to draw attention to the fact that this statement applies merely to such sporadic or accidental cases as might arise independently of or as mere forerunners of an epidemic. No suggestion seems to have been made so far that the Asylums Board, with its present appliances, should undertake the management of a great outbreak of plague, should such a misfortune happen in London; and it must be understood that the obligation which lies upon every local sanitary authority to provide reception houses to receive

**ANNOTATIONS**—*continued.*

"contacts" during the disinfection of their premises remains entirely untouched. It may be well to point out, as bearing upon this subject, that although under ordinary circumstances the various local sanitary authorities enjoy a large amount of independence, of which some of them, it may be said, do not hesitate to take full advantage, the Local Government Board becomes immediately supreme over any part of England which appears to be threatened with, or to be affected by, any formidable epidemic, and may under such circumstances make, and from time to time alter and revoke, regulations for the speedy interment of the dead, for house to house visitation, for the provision of medical aid and accommodation, for the promotion of cleansing, ventilation, and disinfection, and for guarding against the spread of disease; and may by order declare all or any of the regulations so made to be in force within the whole or any part of the district of any sanitary authority, and on the high seas within the jurisdiction of the Lord High Admiral; and further, that the sanitary authorities are bound to do everything that is necessary to carry out these regulations. It will thus be seen that in case of any unfortunate outbreak the central authority is armed with ample powers, and that no pig-headed local authority could interpose its *vis inertiæ* in the way of proper measures being taken for the control of an epidemic.

### The Private Care of Hopeless Lunatics.

THE circumstances of the recent prosecution of Mr. John Hutton, on the one hand for cruelty to his brother, a congenital idiot, a charge which was dismissed, and on the other for housing a lunatic for profit without the necessary licence, for doing which a small fine was inflicted, well illustrates one, at least, of the influences which have of late years led to the very large apparent increase in lunacy throughout the country, for such a case as this shows the risks which anyone runs who attempts to keep an insane relative at home instead of sending him to an institution for the insane. We can have very little doubt that it was a great error in judgment on the part of Mr. Hutton to attempt to retain the care of his idiot brother. The technical point on which the case went against the defendant was that, so far as the £80 to £90 a year which he received for his brother's maintenance can be called profit, he received a lunatic for profit in an unlicensed house, and the fact that, under the circumstances, he should be prosecuted for this, shows the extent to which pressure is being brought to drive into the asylums all such cases. Indeed, when one considers how troublesome a person an idiot or a lunatic of any kind always is to have in a family, how difficult it may be to prove in the case of a working man where such help as the lunatic may be able to give about the house is "profit," and, in the case of others, how impossible it may be to disprove accusations of cruelty brought against the untrained servants who must at times take a share in the supervision of the patient, one wonders that all who are mentally afflicted have not long ago been swept into asylums. That they should be so dealt with is, it may be presumed, the official policy. There is a good deal to be said on both sides. In those stages in which some educational improvement is still possible, everything, of course, that an institution can do should be done to develop such latent mental powers as may exist, and there is no doubt about the great improvement which has been produced by judicious training in some very hopeless-looking cases of congenital idiotcy. But when it becomes merely a question of the patient living out his life, we are not entirely at one with those who would herd all such cases into vast public asylums. As shown by the readiness with which these feeble minds learn to recognise rules, and to submit to discipline, such institutions are no doubt very successful in their way, but, so far as happiness is concerned, asylum idiots do not seem a very happy class.

### Umbrellas on the Field of Battle.

IN a discussion on ambulance service for troops in the field, Surgeon-Major Valentine Matthews, V.M.S.C., recently made a suggestion which seems worthy of attention. It seems unfortunately to be the fact that in some cases it is quite impossible to remove at all speedily the whole of the wounded from the field of battle, and our experience in South Africa has shown us what terrible hardships this involves. Much of the suffering borne by those who have to lie for hours on the field is not, however, due to the wounds by which they are disabled so much as to the blazing sun which hour after hour beats down upon them, or the pouring rain with which they may be drenched. Surgeon-Major Matthews says it would be an advantage if the regimental ambulance waggons were provided with some form of curtain, something of the nature of a light sunblind, readily detachable, which might be supported on light poles, say 2 feet high, and put over the wounded in the ditch or other shelter to which they may have been temporarily brought, or to which they may have crawled. It has also occurred to him, and he says he mentions this at the risk of exciting some ridicule, that some temporary shelter might be obtained by the use of umbrellas. A large umbrella of suitable colour, marked conspicuously with the Geneva Cross, with a flap or curtain, forming an article something between a carriage umbrella and an umbrella tent, might be employed. It should have a spike at the end of the stick, and be pitched under cover not upright but obliquely, anchor-wise, and he believes that in this way shelter might be provided for two or three men, and that by the use of a certain number of such articles some of the suffering due to prolonged exposure to the sun or the weather might be averted. We do not think that the author of this suggestion need apologise. Shelter tents have been much used in other armies, even for those who were not wounded, and as for the umbrella, most of our ideas as to fitness are purely conventional. Of course, the idea of a soldier doing sentry-go under an umbrella is absurd. But is it more absurd than the very familiar sight of a sentry walking up and down Pall Mall in the pouring rain without one? The Boers are without prejudice in military matters, and as soon as they were "commandeered" for the war they are said to have bought up all the umbrellas in the country. They knew their native rains, and, soldiers or no soldiers, they did not see why they should get wet.

